# The Effect of Energy Labelling on Menus and a Social Marketing Campaign on Food-Purchasing Behaviours of University Students

**DOI:** 10.1186/s12889-016-3426-x

**Published:** 2016-08-05

**Authors:** Rajshri Roy, Jack Beattie-Bowers, Siew Min Ang, Stephen Colagiuri, Margaret Allman-Farinelli

**Affiliations:** 1 School of Life and Environmental Sciences, Charles Perkins Centre, The University of Sydney, Level 4, E40, D17, Sydney, NSW Australia; 2 Sydney Medical School, University of Sydney, Sydney, Australia

**Keywords:** Menu labelling, University, Food choices, Young adults

## Abstract

**Background:**

This study assessed the impact of kilojoule (kJ) labelling alone or accompanied by a social marketing campaign on food sales and selection of less energy-dense meals by young adults from a university food outlet.

**Methods:**

There were two kJ labelling intervention phases each of five weeks: (1) kJ labelling alone (2) kJ labels with marketing materials (“8700 kJ campaign”). Food sales of labelled items were tracked during each intervention and five weeks after. Food sales during interventions were also compared with historical sales of foods in the same 10-week period in the previous year. A sub sample of young adults (*n* = 713; aged 19–24) were surveyed during both the interventions to assess awareness, influence, sentiment and anticipated future impact of kJ labels and the social marketing campaign respectively.

**Results:**

There were no differences in sales between the kJ labelling with social marketing and the 5-weeks of labelling before and after. The percentage sale of chicken Caesar burger (3580 kJ, *P* = 0.01), steak and chips (4000 kJ, *P* = 0.02) and the grill burger (5500 kJ, *P* = 0.00) were lower in the year with menu labelling and social marketing campaign. Only 30 % students were initially aware of the kJ labels on the menu but 75 % of students were accepting of kJ labelling, after they were made aware. Respondents viewing the marketing campaign elements and then using kJ values on the menu selected meals with a lower mean energy content; constituting a reduction of 978 kJ (*p* < 0.01) even though the majority claimed that the 8700 kJ campaign would not impact their food choices.

**Conclusions:**

Point-of-purchase energy labelling may be an effective method to encourage better food choices when eating out among young adults. However, further efforts to increase awareness and provide education about energy requirements to prevent weight gain will be needed.

## Background

The prevalence of overweight and obese individuals particularly among young adults has sharply increased in recent years [1], as a result, in part, of changes in the food environment [2]. Research links meals prepared outside the home to higher kilojoule (kJ) consumption, overweight, and obesity in both adults and children [3–7]. Since environmental factors contribute to improving dietary behaviour; interventions need to be planned on a population level [8]. Among the suggested strategies has been increasing the availability of nutrition information for foods eaten and prepared away from home. Fast food chain restaurants in some countries are required to provide energy information on their menu boards [3–7]. Theoretically, provision of energy information at the point-of-purchase (POP) may help improve consumer food choices and limit excess energy intake [9]. However, the limited numbers of studies that have evaluated this approach have produced mixed results [10–14]. Public education campaigns accompanying or preceding restaurant menu labelling may be needed so that individuals understand daily kJ requirements [15].

Limiting energy intake is recommended to reduce obesity and subsequent chronic disease risk [16]. In observance of the need, the New South Wales (NSW) Food Authority in Australia instituted a mandatory kJ menu labelling program. Standard food outlets with more than 20 locations in NSW or above 50 locations nationally, were required to display both kJ contents of food items and signage stating ‘the average adult daily energy intake is 8700 kJ’. The impact and appropriateness of this kJ labelling program were evaluated using intercept interviews with consumers at outlets and found the median kilojoules purchased decreased by 15 % from 3355 kJ to 2836 kJ [17].

To prevent obesity, interventions should be positioned before major weight gain has occurred, and the risk of weight gain is greatest in young adulthood [18]. As young adults are the largest consumers of foods prepared outside the home, such a kJ labelling program might impact on their dietary behaviour and consequently overall diet quality [19]. More than half of all young adults in Australia are engaged in tertiary education settings [20]. Food outlets in these settings are generally exempt from this mandatory labelling as they have less than 20 stores. Tertiary education institutions may, therefore, be ideal settings for measuring the effect of energy labelling interventions on the food-purchasing behaviours of young adults [21].

This study describes the process for implementation of food energy labels and its impact in a demonstration project. The objective was to evaluate the feasibility and effectiveness of the implementation of kJ labelling in an on-campus food outlet as a prelude to a proposed university-wide roll out. The study examined the impact of POP energy information on the sales of different foods and measured customer’s awareness and knowledge about the kJ labels and usage without and with a social marketing campaign. We hypothesised that the sales of highest energy foods would decline via kJ menu labelling. Secondly, we hypothesized the use of social marketing with the kJ labelling would result in greater attention to, and use of, the labels to purchase lower energy menu choices.

## Methods

### Study plan and setting

An advisory group that included the researchers and the staff managing the food service at the university was convened. Study design and implementation was agreed upon by both parties after negotiation. A food outlet where menu decisions could be made quickly and was closest in setting to quick service restaurants was selected for the trial. In this outlet, consumers order food and beverages at the counter after selection from the menu. The menu items from the food outlet were analysed in advance of the project by two independent Accredited Practising Dietitians (APDs) from the standard recipes supplied by the food vendor using FoodWorks software that uses the Australian database of foods (Version 6, 2009, Xyris Software, Spring Hill, QLD, Australia). Food items on the menu were classified as high- and low-energy using established criteria employed in NSW schools [22]. After the dietitians provided nutritional analysis of the menu, the food service vendor decided to change the menu and added some items that they perceived as healthy options such as salads. The new menu items were then analysed by the dietitians. A comparison of seven newly added food items with their removed counterparts was tabled by the researchers. The food outlet vendor asked that beverages, which were served on tap, not be included as they did not have a standardized serving size. The daily special which changed every day at the discretion of the vendor also had to be excluded and these items do not appear on the menu.

**Fig. 1:**
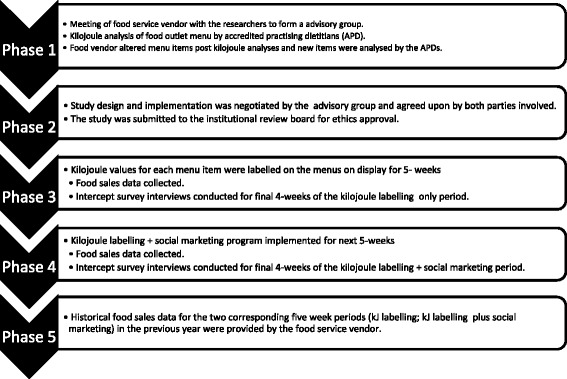
Flow chart of the study design

The study period was planned such that it could be completed in one semester of University that allowed five weeks of kJ labelling only, followed by five weeks with an accompanying social marketing campaign. The data collection was between April and June, 2014. The food service staff members serving were trained about the intervention and two dietitians were available at the outlet during the intervention periods to address any questions from staff. Figure 1 shows a flow chart of the study design.

#### kJ labelling

**Fig. 2:**
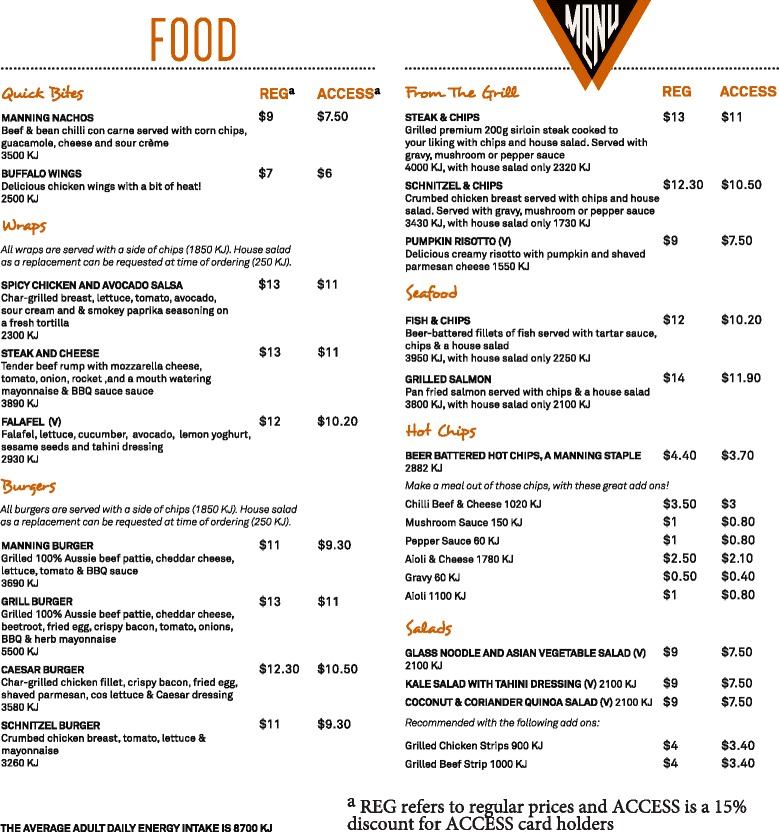
Food outlet menu with energy labels

The NSW Food Authority’s 8700 program guidelines were followed and kJ content was displayed for food items (and a choice of side dish; chips or salads) on the menu (Fig. 2) [23]. Figure 2 shows the cost of the options on the menu. There are two columns for prices such as regular prices and access prices. Access prices are a 15 % discount on menu items for student customers who hold a food vendor loyalty card called the access card. The reference statement about the average adult serve of 8700 kJ was also included on the menu as dictated by the guidelines. Ideally, the kJ value must be adjacent to the price of each item on menus in the same size, colour and font of the price information but the food vendors could only place it in line with the menu item [23]. The kJ labels were same size as the prices and were posted on table menus and on laminated menus at the counter. Item pricing remained unchanged.

#### Social marketing campaign

**Fig. 3:**
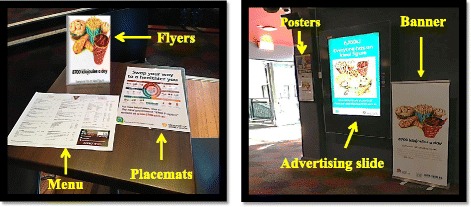
Example social marketing campaign materials in the food outlet

Promotion and marketing campaign resources included a comprehensive website, interactive calculators for consumers to derive personal daily kJ requirements, and info-graphics. These promotional materials were accessed from the NSW government created and regulated website [24] and distributed as coloured A3-sized posters and DL-sized flyers. An advertising slide was designed using materials from the website and displayed on digital screens. A coloured exhibition banner (600 × 1500 mm) and laminated A3-sized placemats were also used. These advertising materials were placed in selected areas within the outlet (Fig. 3). In addition, two dietitians were present during lunch-time hours to answer questions by student consumers during the social marketing campaign. The dietitians stood next to the marketing display banner positioned (Fig. 3).

#### Changes in food sales during the intervention periods

Impact evaluation included observing changes to food purchases using itemized food sales data. Computerized weekly sales data were obtained from the food vendor for all menu items sold during the five weeks of kJ labelling only and for the five weeks of the social marketing campaign. Data for the 5 weeks after the social marketing campaign concluded, while the kJ labelling still remained on the menu, was also provided. The food sales during the social marketing campaign period were compared with the five weeks of kJ labelling only and five weeks following the social marketing campaign. The researchers also collected historical sales data from the 10 corresponding weeks of the same period in the previous year. The sales of the food items that remained unchanged from the previous semester were compared across the corresponding weeks to isolate the effect of energy information on the ordering patterns of customers. ‘Buffalo wings’ and ‘steak and cheese’ sales were excluded from analysis as these were new items which did not replace any previous menu items and therefore there were no corresponding sales in the previous semester. Daily specials or custom orders changed every day at the discretion of the vendor. Such last minute changes made it impossible to analyse the items and therefore, were exempt from the kJ labelling.

#### Customer attitudes, awareness knowledge and use of energy labels

After one week of each of the kJ labelling only and the kJ labelling with social marketing campaign, intercept interviews with students were conducted for the last 4-weeks of each intervention period. Intercept interviews are a type of interview whereby respondents are stopped and invited to be part of the survey at the POP. The interviews were based on questions adapted from those used by the NSW government program with some additional questions [17]. The surveys were implemented during lunch-time hours (1200 h to 1500 h) as the majority of students purchased foods during those hours.

The interview questions have been listed in Table 1. The surveys each consisted of thirteen questions; the first five questions screened and gathered demographic data. The remaining eight questions measured awareness, understanding of kJ content of foods, use of labelling for purchases, impact, perception, attitude, and sentiment about labelling and the campaign. The interviews were conducted at the venue by final year student dietitians and another APD. The participant was invited to be interviewed after their purchase while they are waiting for their buzzer to light up for food collection at the counter. The participant was interviewed where they were seated or standing during lunch time hours i.e. interview period and the interview took ten minutes to complete. Participants were only approached once for the interviews.

**Table 1:** The intercept survey questions asked during kJ labelling, only period and kJ labelling with social marketing period, respectively

Screening and demographic questions	Intercept interview questions	
	kJ Labelling, only	kJ labelling with social marketing campaign
• Are you aged between 19 and 24 years (inclusive) today? • Are you a full time or part time student at this university? • What Faculty are you currently studying in? • What year are you currently enrolled in? • How many times IN A WEEK do you usually eat from this outlet?	• Were you aware of any nutritional information at the outlet? • Where is the observed nutritional information located? • Can you recall the observed specific nutritional information at the outlet? • How does the nutritional information on site influence your food choice? • What did you buy? • Was this choice influenced by the energy labelling? • What do you anticipate will be the impact of these menu labelling changes in Manning Grill? • What do you think about the Kilojoule labelling? Do you: - Like - Dislike	• Recall the location of any recent advertising regarding Kilojoule food and drink labelling • Describe any advertising you had seen or heard. • Look at these marketing stimuli (bar top screen, placemats, poster, and flyers). Have you seen or heard different elements of the KJ labelling marketing campaign? • After viewing all the campaign stimuli, what do you think is the main message of the campaign? • How does the nutritional information (in the marketing campaign stimuli) on site influence your food choice? (Select as many as apply). • What did you buy? • Was this choice influenced by the (marketing campaign stimuli) of energy labelling? • What is your impression of the Kilojoule labelling campaign? (Have you found this campaign informative/necessary/believable/relevant to you?)

#### Participants for the intercept interviews

The selection criteria for participation in the intercept interviews were that the participants needed to be aged between 19–24 years and in second or higher year of study at the university. First-year students were excluded using screening questions as unfamiliarity with the university environment may have affected results. Other demographic information about the participants collected as part of the survey included faculty of study and the number of visits to the outlet in a week.

#### Sample size

We used +/− 5 % margin of error and a 90 % confidence level power calculation to obtain an effect size equivalent to that of relevant past studies and the study aimed to recruit 300 respondents per intervention intercept survey [11, 25]. The study was approved by the University Human Research Ethics Committee (HREC2014/027). Written informed consent was obtained from all participants.

#### Data analysis

Data analyses were conducted using Statistical Product and Service Solutions (Version 22.0, SPSS, Armonk, NY, 2013) [26]. All data were assessed for normality by examining skewness and kurtosis. An ANOVA was conducted to compare the differences in overall sales between menu labelling periods prior, during and after the social marketing campaign. The weekly sales of seven newly added food items were compared with their removed counter parts using the corresponding weekly sales in the preceding year. Results are presented as mean ± standard error. For comparisons with the previous year, the number of food items that remained unchanged throughout was calculated as a percentage of total items sold and the relative change % was compared. Analysis to determine significance between intervention and comparative periods were performed using Mann–Whitney *U* test for non-normally distributed data. Chi-squared tests were used to compare participant demographics, awareness and usage of labels with kJ labelling alone and with kJ labelling + social marketing campaign. An ANOVA with Bonferroni post-hoc test was conducted to compare reported energy consumption by those who were unaware, aware, aware and using (influenced by) labels. Statistical significance was set as *P* < 0.05. Themes from open-ended questions such as noticing kJ labels and their usage, nutrition knowledge and behaviours and support for kJ labels on menus have been synthesized in a narrative form.

## Results

### Menu changes

The kJ values of seven new menu items that were introduced are compared with their original counterparts in Table 2. Three of the seven meals that were added as replacement were actually higher in energy. Table 2 also shows food sales data of new menu items added in the current year compared with food sales of the original counterparts in the equivalent length of time in the preceding year. The proportional sales data showed that the pumpkin risotto which contained less energy than the mushroom and bacon risotto that it replaced sold more (90 %, *P* = 0.00) but the falafel wrap contained more kJs than the burger it replaced and the sales decreased (*P* < 0.02).

**Table 2:** Sales (mean ± SE) of meals removed by the food vendor before kJ labelling compared with the replacement meals over the 10-week period for each, as a percentage of total sales

No kJ labelling and social marketing campaign			kJ labelling and social marketing campaign			Difference	*P* value
Food removed from menu	Energy (kJ)	Mean ± SE	Foods added to menu	Energy (kJ)	Mean ± SE		
Japanese Chicken Burger	2340	24.3 ± 0.27	Chicken, Avocado, Salsa Wrap	2300	33.3 ± 0.36	9.06	0.00^a^
Falafel Burger	2734	17.9 ± 0.12	Falafel Wrap	2930	13.6 ± 0.14	−4.36	0.02^b^
Grilled Fish and Chips	2580	14 ± 0.13	Grilled Salmon	3800	12.9 ± 0.16	−1.16	0.02^b^
Mushroom and Bacon risotto	2488	7.2 ± 0.12	Pumpkin Risotto	1550	28 ± 0.22	20.86	0.00^a^
Caesar Salad	1972	9.2 ± 0.10	Coconut, Coriander & Quinoa Salad	2100	4.1 ± 0.04	−5.17	0.36
Honey Soy Chicken Salad	3080	13.2 ± 0.13	Glass Noodle Asian Salad	2100	5.4 ± 0.05	−7.81	0.41
Salmon Teriyaki Salad	2868	14.2 ± 0.13	Kale & Quinoa Salad	2100	2.8 ± 0.04	−11.42	0.20

Difference represents the change in percentage of sales of foods over the 10 weeks of kJ labelling and social marketing period compared with the corresponding 10 weeks the year before

^a^Significant increase in sales of new labelled items (added post nutritional analysis and pre intervention) compared to previous unlabelled counterparts. *P* < 0.05

^b^Significant decrease in sales of new labelled items (added post nutritional analysis and pre intervention) compared to previous unlabelled counterparts. *P* < 0.05

Analysis to determine significance between intervention and comparative week’s periods performed using Mann–Whitney *U* test for non-normally distributed data

### Food sales results

**Fig. 4:**
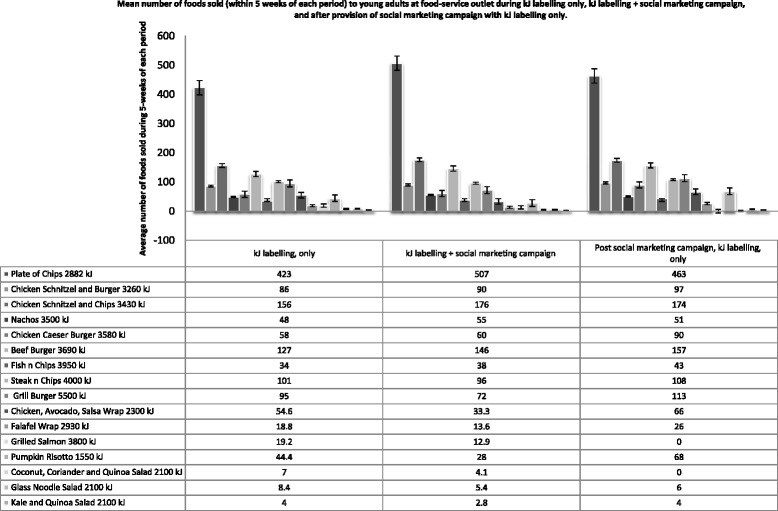
Comparison of mean number of food items sold (mean ± standard error) weekly during 5-weeks of kJ labeling only, during 5-weeks of kJ + social marketing campaign and 5-weeks after the end of social marketing campaign with kJ labels only). Analysis to determine significance between three periods performed using ANOVA; Non-significant change in sales *P* > 0.05

Figure 4 shows the comparison of food items sold during the 5-weeks of kJ labelling only, during the 5-weeks of kJ labelling + social marketing and the 5-weeks after the social marketing campaign ended but labels still in place. Overall there were no changes in sales during and after the social marketing campaign. Table 3 compares the sales of nine items that remained unchanged on the menu during the kJ labelling only, kJ labels + social marketing compared with sales for the same weeks in the preceding year. Mean sales of grill burger (5500 kJ, *P* = 0.05) were lower and chicken schnitzel (3430 kJ, *P* = 0.05) higher with kJ labelling compared with the year before. The sales of chicken Caesar burger (3580 kJ, *P* = 0.01), steak and chips (4000 kJ, *P* = 0.02) and the grill burger (5500 kJ, *P* = 0.00) were lower during the five weeks of social marketing campaign and sales of the chicken schnitzel and chips were higher (3430 kJ, *P* = 0.04) than the corresponding five weeks the year before.

**Table 3:** Sales (mean ± SE) of food items with energy labels; during 5 weeks of each intervention periods compared with corresponding 5 weeks pre-interventions in the previous year

		Baseline period	kJ labelling only period				Baseline period	kJ labelling + social marketing campaign period			
Food	Energy (kJ)	5 weeks pre-kJ labelling in the previous year	5 weeks of kJ labelling only	Difference	Relative change %^a^	*P* value	5 weeks pre-kJ + social marketing campaign in the previous year	5 weeks of kJ + social marketing campaign	Difference	Relative change %^d^	*P* value
<-------------------------------------mean ± standard error------------------------------------->											
Plate of chips	2882	39 ± 0.38	38.5 ± 0.38	−0.51	−1.33	0.15	40.7 ± 0.42	40.1 ± 0.34	−0.61	−1.52	0.29
Chicken Schnitzel Burger	3260	8.4 ± 0.09	7.8 ± 0.08	−0.60	−7.66	0.13	8.2 ± 0.11	7 ± 0.04	−1.27	−18.20	0.11
Chicken Schnitzel & Chips	3430	9.4 ± 0.16	14.2 ± 0.13	4.77	33.65	0.05^b^	9.6 ± 0.06	14.2 ± 0.27	4.61	32.50	0.04^b^
Nachos	3500	4.5 ± 0.04	4.4 ± 0.05	−0.08	−1.94	0.15	5 ± 0.08	4.4 ± 0.06	−0.63	−14.21	0.11
Chicken Caesar Burger	3580	7 ± 0.08	5.3 ± 0.06	−1.66	−31.36	0.06	6.8 ± 0.08	4.6 ± 0.04	−2.27	−49.65	0.01^b^
Manning Burger	3690	12 ± 0.11	11.5 ± 0.12	−0.43	−3.69	0.15	10.7 ± 0.11	11.4 ± 0.06	0.62	5.50	0.21
Fish n Chips	3950	3.3 ± 0.03	3.1 ± 0.03	−0.23	−7.52	0.13	2.8 ± 0.03	3.1 ± 0.08	0.34	11.02	0.21
Steak & Chips	4000	7.7 ± 0.06	8.7 ± 0.08	1.01	11.58	0.13	6.6 ± 0.06	8 ± 0.08	1.42	17.83	0.02^b^
Grill Burger	5500	8.8 ± 0.06	6.5 ± 0.07	−2.26	−34.57	0.05^b^	9.6 ± 0.06	7.4 ± 0.03	−2.23	−30.22	0.00^b^

Difference represents the change in percentage of sales between the intervention and baseline periods (corresponding weeks the year before)

^a^Relative Change % = ((Intervention - Pre)/Intervention)*100

^b^Significant change in sales *P* ≤ 0.05; analysis to determine significance between intervention and comparative weeks periods performed using Mann–Whitney *U* test for non-normally distributed data

### Intercept survey results

Three hundred and fifty-one people completed the intercept interview survey (~75 % response rate) during kJ labelling only. Three hundred and ninety-five respondents were recruited during the kJ labelling and social marketing period survey (64 % response rate). These response rates were estimated from eligible students approached who agreed to take part in the survey versus those who refused; the exact number of students visiting the food outlet during survey time periods could not be determined. Eleven percent of participants chose from un-labelled daily specials and their survey data was excluded from the analysis.

The respondent demographics did not differ significantly between the two surveys (*P* > 0.05). However, most respondents during the first survey were female (64 %) and second-year students (56 %) belonging to the Science faculty (54 %). Percentage of males and females were approximately equal in the second survey. Also in the second survey, respondents were enrolled in either the Faculty of Arts or Science (56 %) and were second-year students (54 %). Overall, respondents reported eating at the outlet less than once per week (41.8 %), followed closely by once per week (36.5 %), twice per week (13.5 %), then more than twice per week (4.1 %, 2.2 %, 1.6 %, 0 %, and 0.3 % respectively).

### Awareness, understanding and use of KJ labels before and during the social marketing campaign

Table 4 shows the results for awareness and use of kJ labels for the two intervention periods. During the period with kJ labels only, 30 % were aware of the labels. However, with the addition of a social marketing campaign, this increased to 51 %. The proportion of respondents reporting that they were aware and influenced by the labels was 5 % but increased with the social marketing campaign to 9 %. Despite not being aware when asked about energy labelling, 75 % said they liked it. A minority of respondents recalled the location of kJ labelling on the menu (26 %), and an even lower proportion recalled the kJ value for their purchase (11 %). All participants who reported being influenced (9 %) said that the labelling helped them be more ‘mindful or informed’ (9 %).

**Table 4:** Intercept survey results of food outlet customers categorized according to intervention

Percentage of students (%)	Menu labelling (*n* = 318) (%)	Mean (SD) energy consumed (kJ)	Social marketing campaign (*n* = 264) (%)	Mean (SD) energy consumed (kJ)	Chi square^a^	Significance^a^
Aware of kJ intervention	30	3353 (732)	51	3811 (1272)^b^	13.82	0.0015
Aware but not influenced by intervention	25	3392 (871)	42	3968 (1382)^c^	9.25	0.0005
Aware and influenced by intervention	5	3313 (966)	9	2833 (1182)^b, c^	5.99	0.0604

^a^Chi-squared tests for differences in proportions of awareness and influence of intervention

ANOVA with Bon-ferroni post-hoc test

^b^Reported mean kJ consumed between respondents who recalled campaign and used labels and respondents who did not recall campaign: *p*-value < 0.01, 99 % 978 kJ CI (129.7, 1721.6)

^c^Reported mean kJ consumed between respondents who recalled campaign elements but differed in reported label use *p*-value < 0.01, 99 % 1135 kJ CI (126.9, 1796.2)

Respondents who noticed both the campaign and used kJ labels (influenced by) for purchases bought an average of 978 kJ (99 % CI 129.7, 1721.6) less than respondents who had not noticed the campaign *P* <0.01 (see Table 4). The energy purchased between respondents who recalled campaign elements and who reported label use and those who recalled campaign elements but did not report label use, were also significantly different (1135 kJ difference) (99 % CI 126.9, 1796.2, *P* <0.01). Males and females were equally likely to recall and/or use campaign elements for purchases (*χ*2 (4) = 4.108, *p* > 0.63). Males bought 725 kJ on average more than females (99 % CI (82.6, 661.9, *P* <0.05).

With respect to perceived impact assessed by open-ended questions, respondents believed that the menu labelling would have no impact because “people will eat what they want to eat” or “people who [already] count calories would pay attention to the menu labels” and that “people have already made a choice before arriving at the food outlet”. Those respondents who believed that the menu labelling will have a positive impact stated “people would pay attention”, they would “be healthier” and that it would be “a good thing for students”. However, they also added that the impact would be small. Ninety percent of respondents were able to infer the correct health message from the campaign elements and 40 % of respondents anticipated a positive change in health behaviour with ordering a lower kJ item being the most common change. The campaign received a positive impression from respondents (96 %).

## Discussion

The objective sales data demonstrated no appreciable change in sales of menu items with social marketing of the kilojoule program. However, the cross-sectional survey findings suggest that kJ labels + social marketing campaign increase consumer awareness and had a small impact on self-reported use of kJ labels on menus and lead to lower energy choices This is consistent with the literature that suggests it is not uncommon for patrons to report awareness and self-reported use of labels but still purchase high-energy foods [27].

A recent systematic literature review reported the importance of easy visible calorie labelling for obesity prevention, and it appears an attractive strategy to many young adults [28]. It appears that to be effective, calorie labels must be large and prominent, and the source of information believed to be from a credible authoritative source [29]. The kJ labels applied in this study were the same size as the prices in keeping with the regulations but they might be too small and insignificant. Our social marketing campaign provided an opportunity to explain the source and believability of the information. A social marketing campaign with kJ labelling theoretically is supposed to increase the effect of labelling at POP [24]. The location and elements of campaign materials installed were consistent with recommendations for advertising materials designed for attracting attention and creating awareness among university students [30, 31]. As expected an increase in awareness of energy label was found [11, 32, 33]. However, food-purchasing behaviours are influenced by environmental, personal and label related factors and it is the interplay of these factors that will determine whether and how menu labelling affects dietary behaviour of young adults [34].

The intercept interviews provide modest support for the hypothesis that nutrition information in the form of kJ labels combined with a social marketing campaign can influence awareness of kJ labelling and increase the selection of low-energy menu items. Despite the small effects, expansion of the kJ labelling program to all outlets was supported by the high ‘like’ response, even when ‘awareness’ was much lower. The findings of the social marketing campaign are similar to population awareness (51 %) of energy labelling in the NSW food authority initiative (50 %) [17]. In comparison, in a study by Nikolaou et al., where they employed much more prominent energy-labels, 56 % reported using the energy-labels, 97 % of them used energy labels to make lower-calorie choices [29]. Unlike most other studies of this kind, females were no more likely to use kJ labels than males [35]. Participants who recalled campaign elements and used kJ labels bought significantly lower kJ compared to other respondents. The proportion of reported label users was small but only assessing impact on one occasion does not allow the observance of changes in responses that may occur over time. Follow-up studies should therefore be conducted to reassess the impact of the campaign on consumer food choice over time.

A comparison of proportional sales during the kJ labelling intervention and sales of menu items in the previous year did indicate a fall in sales of some of the highest energy items. In addition, while the individual impact may be small, labelling might encourage food outlets to reformulate their foods and make the food supply healthy [36]. However, it is suggested that they work closely with nutritional professionals because as shown in this study what was perceived as lower energy by the food service vendor was sometimes higher in energy than the item it replaced.

The reported intercept interview results of anticipated behaviour change as an impact of the kJ labels were similar to the evaluation results of the NSW health menu labelling initiative [17]. The results from the intercept interviews, however, were not reflected in the food sales results unlike some previous studies [17, 18, 28, 29]. The Australian public may be more familiar with the term “calorie” and as one study demonstrated, many people expressed a lack of confidence about their understanding of kilojoules [37]. However, since state governments in Australia currently require food retailers to display kilojoule information at POP and on the nutrition information panels on packaged foods, familiarity is growing [17, 38–42]. This challenge was addressed in this study, by developing the social marketing campaign that encouraged consumers to notice the kilojoule values displayed on menus in the food outlet, read flyers; view info graphics and display banners, and interact with the dietitians in the outlet. Future studies could replicate this pilot study and observe if the labels and the campaign have a significant impact on food sales over a longer period of exposure to the campaign.

This study focused efforts on the preventative measure for obesity, thus assessing perceived susceptibility to obesity might influence the results and predict behaviour change [43]. The participants were divided when asked about the influence of kJ labels on food purchase. The intercept interviews did not assess whether the food choices were representative of usual diet patterns and this discrepancy could be due to the meals at the chosen food outlet being regarded as “treats” by the participants and bias the food choice towards higher energy foods. The majority of students purchased at this food outlet no more than weekly, however about 15 % of the students purchased at least twice per week at this outlet and choosing lower kJ options would be more important with these high frequency users. Taste was the primary reason given by participants for food selection regardless of kJ labelling. Young adults, therefore, have a low perceived susceptibility towards developing obesity, low priority for eating well, are concerned with taste and consequently low motivation to change current health behaviours [31, 44].

### Strengths and limitations

The intervention was uniquely targeted in order to address the needs of the population, i.e. young adults (19–24 years). The campaign focused on the urgency of prevention of weight gain by maintaining the energy balance. The intercept interview survey drew a large audience with high response rate. We included the objective measures of food sales data rather than just self-reported purchases. This study also has several limitations. The menu changed between the baseline and the interventions in the following year. This was not within the researchers’ control and is an obvious limitation as the change in comparative food sales data before-and after kJ labelling could be confounded by changes in the menu items itself. However, this is the reality of pragmatic research in a university setting. The respondents were all university students with a fairly similar socio-economic status, literacy and numeracy levels. The data distribution was skewed towards students from the faculty of Science and Arts, potentially limiting the generalizability of the study. Self-reported data regarding campaign awareness and dietary intake could have led to response bias. The study sample was primarily female, introducing potential gender bias. Also, there could have been selection bias associated with cost as all food items did not have identical pricing. The short duration of the interventions may alter the effectiveness in the long-term and there is no follow-up as yet. Body mass index (BMI) was not assessed, and the inclusion of lean respondents who are nonchalant about energy balance might lower the intervention efficacy among consumers who could benefit from it. Furthermore, intercept interviews are often associated with social desirability bias which may affect study validity.

## Conclusion and future implications

This is a tertiary education setting based energy labelling study that shows that POP energy labelling intervention is well accepted but not influential on diet choices. Awareness of labelling can be enhanced by effective social marketing. However, the majority of respondents anticipated that the kJ labelling and the social marketing campaign would have no effect on their food choices. Young adults may have low perceived susceptibility to weight gain and a low motivation to change current behaviour. There are important lessons around the nonchalance of most young people who do not perceive a problem and the results point to a need for more incisive interventions. It would appear that in addition to menu labelling, nutrition education and further exploration as to why around almost half the participants failed to notice and therefore use the kJ labels might be needed.

## Abbreviations

APD, accredited practising dietitian; BMI, body mass index; kJ, kilojoule; NSW, new south wales; POP, point-of-purchase
